# Multifocal EBV-associated smooth muscle tumors in a patient with cytomegalovirus infection after liver transplantation: a case report from Shiraz, Iran

**DOI:** 10.1186/s13000-021-01180-6

**Published:** 2022-01-07

**Authors:** Mohammad Hossein Anbardar, Neda Soleimani, Dornaz Safavi, Ahad Eshraghian, Abbas Ayoub

**Affiliations:** 1 Department of pathology, Shiraz Medical School, Shiraz University of Medical Sciences, Shiraz, Iran; 2 Department of pathology, Shiraz Transplant Center, Abu Ali Sina Hospital, Shiraz University of Medical Sciences, Shiraz, Iran; 3 Abu Ali Sina hospital, Shiraz, Iran

**Keywords:** EBV, CMV, Smooth muscle tumor, Post-transplant smooth muscle tumor, EBER

## Abstract

**Introduction:**

Immunodeficient patients, including the recipients of solid organs, exhibit an increase in the incidence of neoplasms. Post-transplant smooth muscle tumor (PTSMT) is a distinct and infrequent entity of these groups of neoplasms. Epstein–Barr virus (EBV) is considered to be involved in the etiology of this neoplasm.

**Case report:**

A 28-year-old man who underwent liver transplantation presented with abdominal pain and diarrhea for several months. He had a history of resistant systemic cytomegalovirus (CMV) infection after transplantation. Radiologic evaluation and colonoscopy revealed multiple liver, spleen, lung, and colon lesions. Microscopic assessment of colon and liver lesions using IHC study were in favor of spindle cell proliferation with mild atypia and a mild increase in mitotic rate without any necrosis, with features of smooth muscle tumor. Considering the transplantation history, EBER chromogenic in situ hybridization (CISH) study on paraffin blocks was requested, which demonstrated EBV RNA in tumor cell nuclei, suggesting EBV-associated smooth muscle tumor. In addition, PCR for CMV on paraffin blocks was positive. PCR for EBV and CMV viremia were negative. The dosage of immunosuppressive agents was reduced, and currently, he is being followed, with slow expansion in the size of the lesions.

**Conclusion:**

Although the incidence of post-transplant smooth muscle tumors (PTSMTs) is low, it should be remained in the differential diagnosis in post-transplantation patients, especially dealing with multifocal tumors. As strong stimulant for smooth muscle tumors, close follow-up and screening for EBV and CMV infection and early treatment at the time of diagnosis are recommended to avoid these virus-induced tumors.

## Introduction

Immunodeficient patients exhibit the increased incidence of neoplasms, whether the immunodeficiency is in the context of genetic disorders, acquired immunodeficiency syndrome (AIDS), or rigorous immunosuppressive therapy for organ transplantation [1, 2]. The development of neoplasms is a well-known complication after solid-organ transplantation. Among these neoplasms, post-transplant smooth muscle tumor (PTSMT) is a distinct and infrequent entity, which may appear in the peripheral soft tissue, intracranial space, and visceral sites. Tumor multiplicity is also common. Epstein–Barr virus (EBV) is hypothesized to play a role in the etiology of this neoplasm [2–5]. Herein, we present a new case of multifocal EBV-associated, cytomegalovirus (CMV) correlated PTSMT occurring in the gastrointestinal tract, liver, and lungs after liver transplantation.

## Case report

A 28-year-old man, presented with abdominal pain and diarrhea for several months. Two years ago, he underwent liver transplantation from a deceased donor due to acute liver failure. During routine post-transplantation follow-up visits, he developed subclinical hypothyroidism and one episode of acute cell-mediated rejection, which were controlled by the administration of levothyroxine (100 μg/day) and immunosuppressive pulse therapy, respectively. He also had resistant systemic CMV infection. He had about 10 kg weight loss during the last 6 months. There was no history of fever or lymphadenopathy. Other past medical histories before transplantation and family history were not significant. He was taking prednisolone, Prograf, and CellCept. On physical examination, the vital signs were stable (blood pressure: 110/70 mmHg, the pulse rate: 68/min, temperature: 36.9 °C), but he had mild abdominal tenderness. The main laboratory data are shown in Table 1.

**Table 1:** The main laboratory data of the patient

Parameter (unit)	Result	Reference range
**WBC(× 10**^**3**^ **/μL)**	4.9 (with 20% atypical lymphocytes)	4.5–11
**HB(g/dL)**	12	14-18
**MCV (fl)**	72.15	80–96
**PLT(× 10**^**3**^ **/μL)**	185	150–450
**ALT (IU/L)**	23	3-40
**AST (IU/L)**	20	3-40
**ALP (IU/L)**	112	80–306
**Total Bilirubin (mg/dL)**	0.95	0. 2-1
**Direct Bilirubin (mg/dL)**	0.3	0. 1-0.3
**BUN (mg/dl)**	14	6-20
**Creatinine (mg/dl)**	1.2	0. 5-1.3
**Fasting blood sugar (mg/dl)**	114	70–99
**HBS Ag**	Non- reactive	Non- reactive
**HCV Ab**	Non- reactive	Non- reactive
**HIV Ab**	Non- reactive	Non- reactive
**CMVviremia PCR**	Positive	–
**AFP (ng/ml)**	4.23	0.89–8.78
**CA19–9**	14.82	< 37

**Fig. 1:**
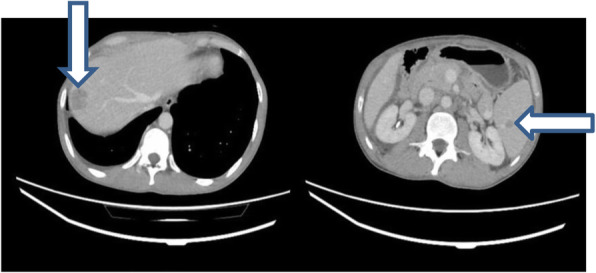
Spiral CT scan showed well-defined solid lesions in the liver (vertical arrow) and spleen (horizontal arrow)

**Fig. 2:**
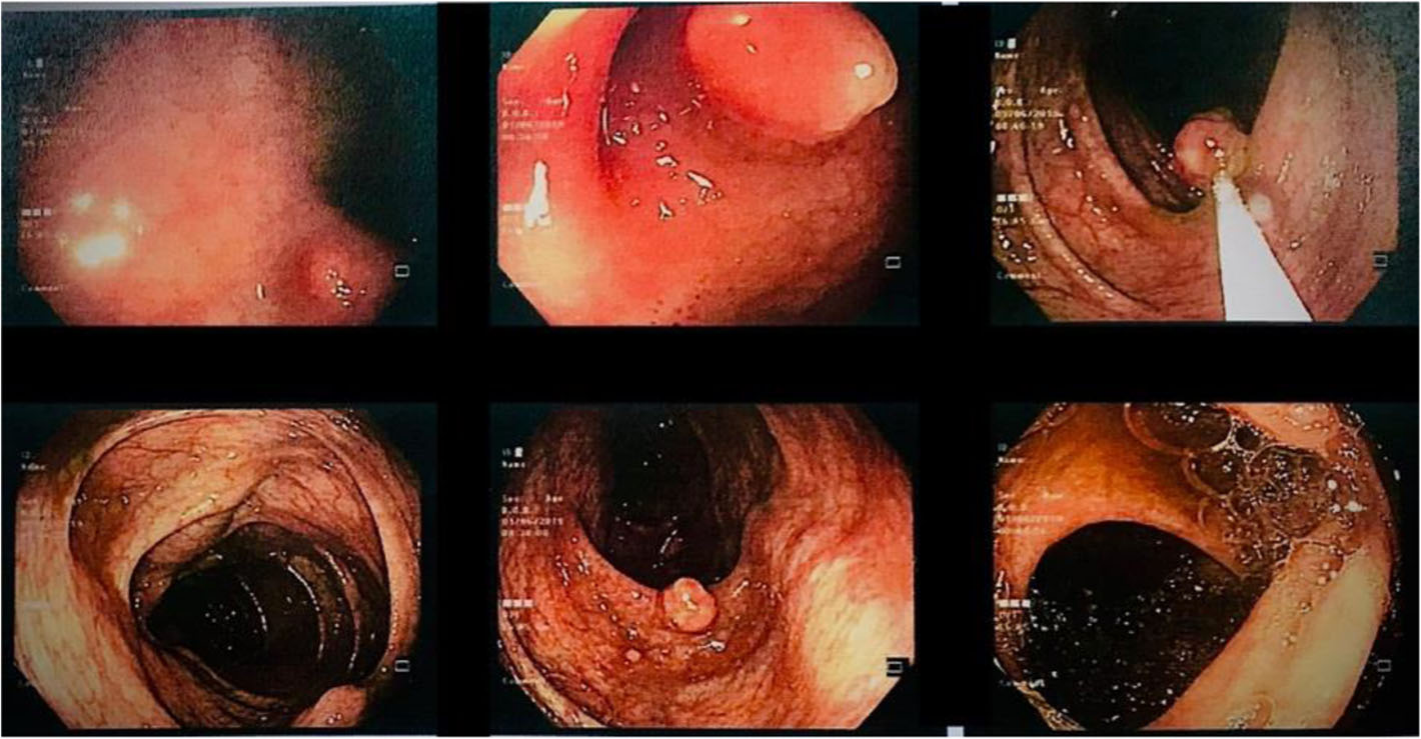
Colonoscopy showed multiple submucosal polypoid lesions

**Fig. 3:**
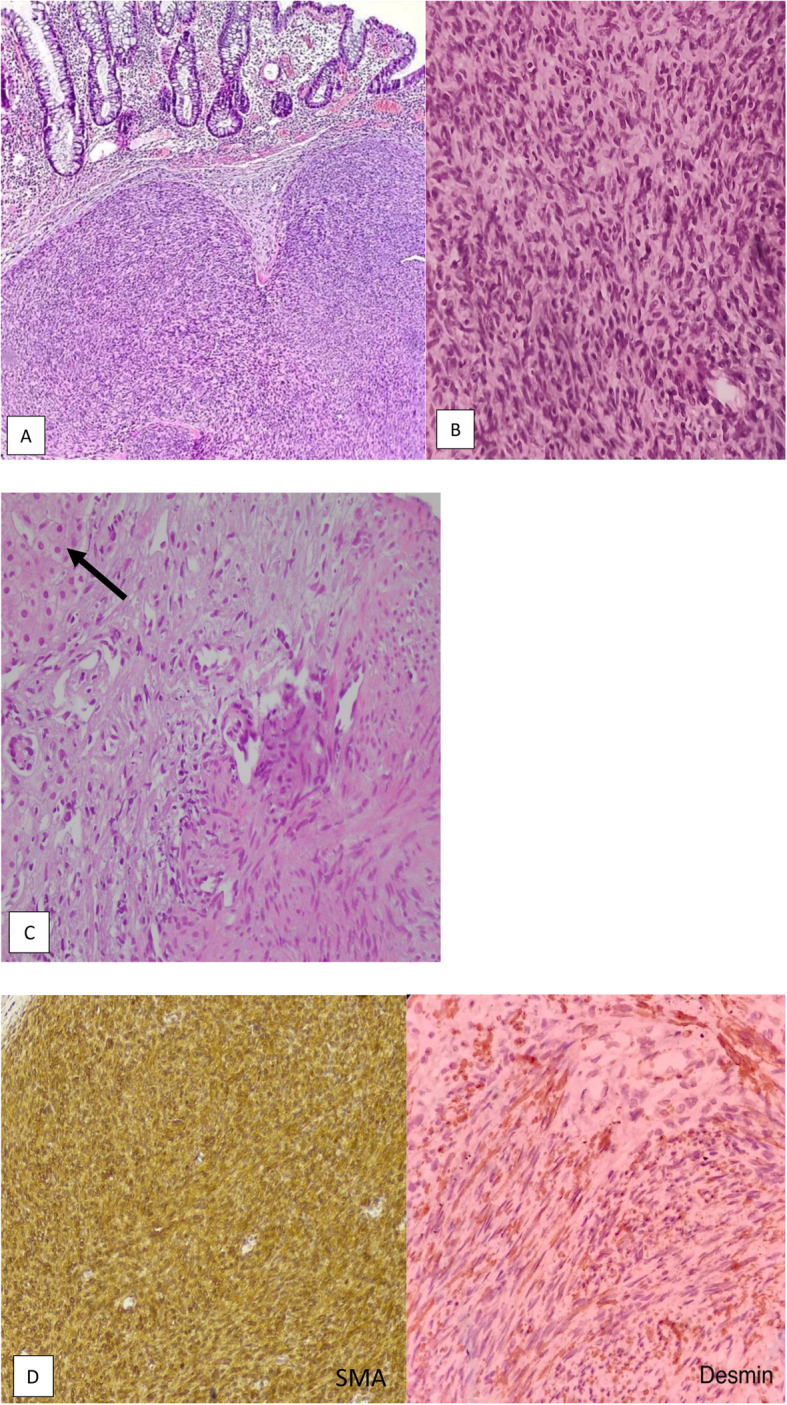
Microscopic view of colon polyps, (**A**)(H&E × 100), (**B**)(H&E × 400), and the liver mass (**C**)(H&E × 40) show intersecting fascicles of spindle cells with elongated nuclei, eosinophilic cytoplasms, mild atypia, and low mitotic activity. There is a thin rim of non-neoplastic liver tissue (white arrow). **D** The neoplastic cells are reactive for SMA and desmin

**Fig. 4:**
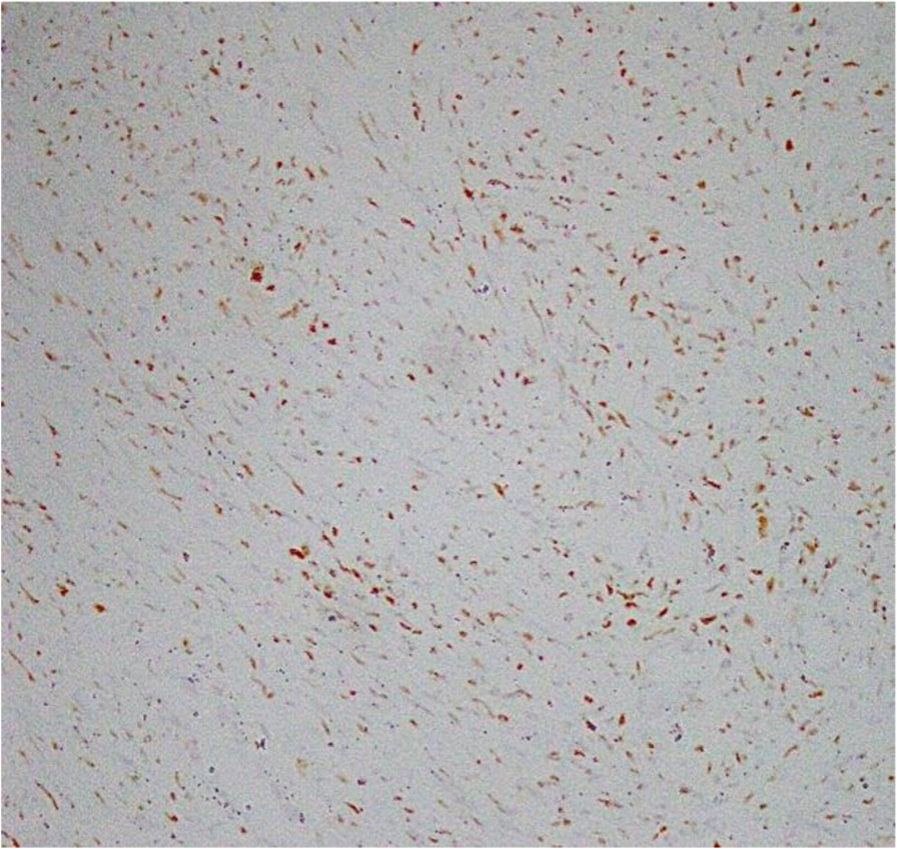
Epstein–Barr virus (EBV)-encoded RNA (EBER) positivity of tumor cells by chromogenic in-situ hybridization (CISH) study

Abdominopelvic ultrasonography revealed a solid liver mass. Spiral chest and abdominopelvic CT scan with contrast were done showing the mentioned lesion with ring enhancement M: 22 × 16 mm in the lateral aspect of segment VIII, in addition to a solid lesion in spleen and multiple small lesions in lower lobes of both lungs measuring 5 to 20 mm (Fig. 1). Colonoscopy was done, which revealed multiple small raised polypoid lesions throughout the rectum and colon (Fig. 2). Microscopic evaluation of the colon mucosa biopsy using immunohistochemistry (IHC) study was in favor of spindle cell neoplasm with high proliferative index. He underwent a right hemicolectomy. The gross morphologic evaluation showed multiple small submucosal polypoid lesions. A microscopic study showed intersecting fascicles of monotonous spindle cells with indistinct borders, cigar shaped nuclei, eosinophilic cytoplasm, mild atypia, and a mild increase in mitotic rate (Ki-67: 15%) without any necrosis with features of smooth muscle tumor (Fig. 3A, B). Trucut biopsy of the liver mass was also in favor of smooth muscle tumor (Fig. 3C). IHC study with desmin and smooth muscle actin (SMA) confirmed the diagnosis (Fig. 3D). According to transplantation history and immune deficiency status of the patient and considering the possibility of EBV-associated smooth muscle tumor, an EBER CISH study on paraffin block of colon lesions was requested, which demonstrated EBV RNA in tumor cell nuclei and immunoblasts of the adjacent lymph node, suggesting EBV-associated smooth muscle tumor (Fig. 4). Although there was no serologic or molecular test for EBV in the patient’s documents, he had a history of prolonged and resistant CMV infection. Thus, polymerase chain reaction (PCR) for CMV was requested on paraffin block of the colon lesions, which also showed positive results (40,000 copies/ml). PCR for EBV and CMV viremia were negative. The dosage of immunosuppressive agents was reduced, and currently, he is being followed, with slow expansion in the size of the lesions.

The written informed consent was requested and obtained by the patient for publishing the case report and the publication of the accompanying images. Also, our institutional approval was not required to publish the case details.

## Discussion

Immunosuppressed patients are susceptible to a variety of neoplasms, whether the immunodeficiency is due to genetic disorders, AIDS, or immunosuppressive therapy for organ transplantation. Lymphoma and cutaneous malignancies are the most commonly reported neoplasms. PTSMT is also a rare entity in this field. This was first expressed by Pritzker et al. in 1970, and in 1995 Lee et al. noticed the role of the EBV in tumorigenesis [1–3]. Conventionally, the condition has been more frequent in AIDS patients, and a small number of cases have been solid organ transplant recipients [3]. The vast majority of PTSMTs appear after kidney transplantation (60%), and the liver is the most common location in both the pediatric and the adult patients. Lungs, lymph nodes, adrenal gland, spleen, heart, kidney, and less often, the central nervous system may also be involved [5, 6]. The precise incidence is not known, but they are reported to arise in less than 1% of the immunodeficient population. The average time it takes for this tumor to appear following an organ transplantation is 4 years [6–8].

The pathogenesis of EBV-SMT is not well recognized. EBV is a DNA herpes virus with a potential to immortalize infected cells. It has traditionally been related to the pathogenesis of Burkitt lymphoma, nasopharyngeal carcinoma, gastric carcinoma, and other B-cell lymphomas in immunodeficient patients [6, 9, 10]. Since EBV-PTSMT can be developed in various organs, some authors hypothesized that SMT may originate from smooth muscle cells of the blood vessel wall. Immunosuppression may permit an abnormal pass of EBV into smooth muscle cells, which could direct to a latent infection and consequent neoplasm formation altered by cytogenetic incidences [11, 12].

Greater than 50% of patients with EBV-SMT present with multiple tumors, and this multifocal involvement appears to be the result of multiple infectious events rather than metastases from a single neoplastic site. EBV-SMTs are often slow-growing and invading only locally. Despite the common multifocal presentation of EBV-SMTs, they are often not fatal [9, 12, 13].

Smooth muscle tumors have a wide range of clinical behavior and pathologic features. Spindle cells are organized in a storiform pattern with scattered small T lymphocytes. These tumoral cells are positive for smooth muscle markers, but negative for angiogenic markers (such as CD31 and CD34) and CD117. PTSMTS can be broadly classified into those with obvious malignant signs of leiomyosarcoma, and ‘borderline’ cases with little pleomorphism, cellular crowding, and mitotic activity with ‘uncertain biological potential’ [1, 3, 5, 14].

The most reliable methods for detection of EBV within tumor cells are demonstration of EBV RNA by in situ hybridization (ISH) and PCR [2, 3].

Although serology confirms previous EBV infection, it has little bearing on diagnosis, recurrence monitoring, or disease burden [9].

Surgical excision with negative margins, especially in unifocal lesions, is frequently curative [5, 9, 15]. Reducing immunosuppressive medication may allow EBV-specific cytotoxic T-cell responses to proliferate, but the risk of graft rejection must also be considered. Incorporating antiviral medicine that reduce EBV viral load and cyclosporine by sirolimus (as an oncogenesis inhibitor) improve disease control and may lead to better outcomes. Antiviral therapy, chemotherapy, and radiotherapy have not been demonstrated to be effective in all cases, but they may be useful in patients with unresectable neoplasms [5, 9].

Krenzlin et al. recently discovered that mouse CMV can be reactivated in perivascular, intratumoral pericytes of glioblastoma, as well as its involvement in tumor promotion [16]. Likewise, human CMV has also been identified as a cancer-causing virus by demonstrating its presence in > 90% of common tumor types such as breast cancer, while being absent in surrounding normal tissue. The biological features of this virus indicate that it is an oncogenic virus. Several Human CMV-encoded proteins have biological properties that are linked to cellular transformation and tumor progression [17, 18]. Human CMV infection has a broad cellular tropism, and it can be found in tumor epithelial cells, macrophages, endothelial cells and even tumor stroma cells [19]. Having invaded many cell types in tumor tissues, CMV can cause all the ten proposed hallmarks of cancer including sustaining proliferative signaling, evading growth suppressors, resisting cell death, enabling replicative immortality, inducing angiogenesis, activating invasion and metastasis, genome instability, reprogramming of energy metabolism, and evading immune destruction [17, 20–22]. Furthermore, the level of CMV infection has a negative relationship with the positive disease outcome, and treatment with antiviral medication in CMV positive cancer patients point to improved prognosis and potentially represents a new valuable anti-cancer approach [17, 23, 24].

Using recent detection methods (ISH, PCR, electron microscopy, DNA and RNA sequencing, immunostaining of tissue specimens, and flow cytometry) some research groups have discovered a high prevalence of CMV in breast, colon, and prostate cancer, rhabdomyosarcoma, hepatocellular cancer, salivary gland tumors, neuroblastoma and brain tumors (medulloblastoma and glioblastoma) [23, 25–30].

Our case was a post-liver transplant patient with consequent colon polyposis, liver and lung lesions, which histologically were compatible with EBV-associated smooth muscle tumor. Despite the fact that he was not checked for EBV infection during post-transplantation follow-up, he had recurrent and resistant systemic CMV infection. Generally, according to previous investigations, CMV infection should also be considered as a stimulator for smooth muscle proliferation besides EBV infection.

## Conclusion

The incidence of PTSMTs is low. However, it should be remained in the differential diagnosis in post-transplantation patients, especially dealing with multifocal tumors. As strong stimulators for smooth muscle tumors, close follow-up and screening for EBV and CMV infection and on time treatment at the time of diagnosis are suggested to prevent these virus-induced tumors.

## Data Availability

All data generated or analysed during this study are included in this published article.

## Abbreviations

AIDS: Acquired immune deficiency syndrome
PTSMT: Post-transplant smooth muscle tumor
EBV: Epstein–Barr virus
CMV: Cytomegalovirus
IHC: Immunohistochemistry
SMA: Smooth muscle actin
CISH: Chromogenic in situ hybridization
PCR: Polymerase chain reaction
ISH: In situ hybridization
